# Trends in HPV cervical and seroprevalence and associations between oral and genital infection and serum antibodies in NHANES 2003–2012

**DOI:** 10.1186/s12879-015-1314-0

**Published:** 2015-12-21

**Authors:** Andrew F. Brouwer, Marisa C. Eisenberg, Thomas E. Carey, Rafael Meza

**Affiliations:** Department of Epidemiology, University of Michigan, Ann Arbor, 48109 USA; Department of Mathematics, University of Michigan, Ann Arbor, 48109 USA; Department of Otorhinolaryngology, University of Michigan, Ann Arbor, 48109 USA

**Keywords:** Human papillomavirus, Prevalence, Type-concordant infection, NHANES, HPV vaccine, Age-Period-Cohort modeling

## Abstract

**Background:**

HPV infects multiple sites in the epithelium, including the genitals and oral cavity. The relation between genital and oral infections and serum antibodies can help explain the natural history and epidemiology of HPV.

**Methods:**

We analyzed HPV data from NHANES derived from self-collected vaginal swabs (women ages 14–59, 2003–12), oral rinses (men and women 14–69, 2009–12), and serum (men and women 14-59, 2003–10).

**Results:**

Type-concordance of cervicogenital and oral infections in women was found to vary widely by age. Prevalence of oral infections with type-concordant antibodies was low but varied by sex: 0.2 % (95 % CI 0.0–0.8) for women vs 0.8 % (95 % CI 0.4–1.3) for men. Vaccination was associated with a reduced risk of cervicogenital infection for vaccine genotypes among ages 14–17 (0.2 (95 % CI 0.1–0.8)) and 18–24 (0.2 (95 % CI 0.1–0.3). Seroprevalence trends in women showed a dramatic increase for recent birth cohorts, likely due to vaccination. By contrast, trends for men remained relatively constant. Age-specific cervicogenital prevalence showed a consistent peak in the late teens and twenties. Relative cervicogenital prevalence has largely been decreasing since the 1940–50 birth cohort.

**Conclusions:**

There are complex patterns in HPV prevalence trends and type-concordance across infection sites and serum antibodies. A multisite sampling scheme is needed to better understand the epidemiology and natural history of HPV.

**Electronic supplementary material:**

The online version of this article (doi:10.1186/s12879-015-1314-0) contains supplementary material, which is available to authorized users.

## Background

The human papillomavirus (HPV) infects multiple mucosal sites in the epithelium and is the etiological agent for over 90 % of anogenital cancers and an increasing fraction of oropharyngeal cancers [1]. Although the progression from cervicogenital HPV infection to cancer has been well documented because of access to tissue during gynecological exams, very little is understood about the progression to cancer in the head and neck. Indeed, although testing for HPV at cervicogenital sites has been standard for some time, characterization of oral prevalence has only recently begun. Further, the association between infection at different sites and their relation to seroconversion is not well characterized. Ideally, a single test, such as for seropositivity of certain HPV strains, could act as a biomarker for the risk of genital and oropharyngeal cancer [2].

There are many strains of HPV, and these are typically classified according to their oncogenic risk. Genotypes 16, 18, 31, 33, 35, 39, 45, 51, 52, 56, 58, and 59 (Group 1) are known to cause cancer, genotype 68 (Group 2A) probably causes cancer, and genotypes 26, 30, 34, 53, 66, 67, 69, 70, 73, 82, 85, 97 (Group 2B) are possibiliy carcinogenic [3, 4]. HPV infection is associated with nearly every cervical cancer, 90 % of anal cancers, 60–90 % of some subsites of head and neck cancers, and 40 % of other genital cancers [1, 5]. HPV 16 causes about 70 % of genital cancers and together 16 and 18 are responsible for 90 % [1]. HPV 6 and 11 cause 90 % of anogenital warts [1]. HPV 16 is also found in 90 % of HPV-positive squamous cell carcinomas (SCCs) in the head and neck [6]. Most HPV infections clear within a year or two [7–10], but some infections may persist for decades and result in oncogenesis.

Vaccines have been developed to target certain strains of HPV. Three vaccines are currently approved by the FDA: GlaxoSmithKline Biologicals’s bivalent (16, 18) Cervarix, and Merck’s quadrivalent (6, 11, 16, 18) Gardasil and Merck’s nonavalent (6, 11, 16, 18, 31, 33, 45, 52, 58) Gardasil 9 [11]. Vaccination against HPV is targeted at females ages 11–12 but is recommended in the US for both men and women with minimal sexual activity under the age of 26 [12]. Vaccine coverage in the US has been low, though increasing, especially among boys [13].

Few studies have thus far considered multiple-site concurrence or type-concordance. Steinau et al. [14] reported that, in the 2009–10 National Health and Nutrition Examination Survey (NHANES), oral HPV infection was five-fold higher in women ages 18–59 with a current cervicogenital infection, and that type-specific concordance was low. The Hawaii cohort study reported a relative risk of 20.5 for acquiring a type-concordant anal infection after a cervicogenital infection and a relative risk of 8.8 for acquiring a type-concordant cervicogenital infection after an anal infection [15]. Genital, anal, and serum data from the HPV in Men (HIM) study paint a complicated picture in which serum antibody levels and seroprevalence of certain HPV types are “higher in men with corresponding anal HPV infection, regardless of genital coinfection, compared with men with genital HPV infection alone” [16] but that seroconversion rates were higher following gential infection than anal infection [17]. No seroconversion was detected following oral infections, though the number of oral infections was too small (*n*=3) to be especially informative [17]. To our knowledge, no other studies have been published considering seroconversion due to oral infections.

Like many sexually transmitted diseases, prevalence of HPV varies widely by demographic group in the US, possibly because of sexual assortativity and differences in sexual behavior patterns. Genital prevalence among non-Hispanic blacks, for instance, is significantly higher than for non-Hispanic whites and Hispanics, and oral prevalence among non-Hispanic blacks and Hispanics is higher than for non-Hispanic whites [14]. Further, prevalence varies significantly with age. However, no attempt has yet been made to disentangle the effects of age, birth cohort, and time period for trends in HPV prevalence. One way to differentiate these effects is by the use of age–period–cohort (APC) models [18–21]. APC models have been used for myriad public health issues including mortality [22], smoking histories [23], and the incidence of several cancers [24–29].

In this paper, we analyze three HPV outcomes: infection at cervicogenital sites (women only), infection at oral sites (men and women), and presence of HPV antibodies in serum (men and women), which is an indication of either a previous infection at any site or of vaccination. We analyze associations between these outcomes by demographic group and model temporal trends in cervicogenital infections and seroprevalence in men and women.

## Methods

### Data

The CDC’s National Center for Health Statistics (NCHS) administers NHANES, a series of studies combining physical examinations in a mobile examination center (MEC) and interviews (both in-home and audio-assisted in-MEC) of a representative sample of the non-institutionalized, civilian population of the US. Each survey is conducted over a two-year period and is used to assess the health and nutritional well-being of the US [30]. Study design, weighting, and collection of samples have been previously described [31–34]. The NHANES survey 2003–12 was approved by the NCHS Research Ethics Review Board (2003–04: protocol #98–12; 2005–10: protocol #2005–06; 2011–12: protocol #2011–17), and documented consent was obtained from all participants.

Self-collected vaginal swabs were analyzed for women ages 14–59 in five NHANES iterations (2003–12) for 37 HPV genotypes, namely 6, 11, 16, 18, 26, 31, 33, 35, 39, 40, 42, 45, 51, 52, 53, 54, 55 (a subtype of 44), 56, 58, 59, 61, 62, 64 (a subtype of 34), 66, 67, 68, 69, 70, 71, 72, 73, 81, 82, 82 subtype IS39, 83, 84, and 89 (formerly CP6108), with the Roche Linear Array. Serum samples were collected and typed using Luminex Multiplex Assay for HPV types 6, 11, 16, and 18 for both men and women ages 14–59 for the same surveys, although the 2011–2012 data is not yet available. Oral rinses were administered to both men and women ages 14–69 in the 2009–10 and 2011–12 surveys and tested for the above 37 genotypes using PCR. The numbers of individuals sampled by demographic group are reported in Table 1. Restricted data (ages 14–17) was accessed through agreement with the NCHS Reseach Data Center. The analyses of this study were approved by the University of Michigan Medical School IRB (HUM00090326).

**Table 1:** Numbers of people conclusively tested for HPV or HPV antibodies at each site in the 2003–04, 2005–06, 2007–08, 2009–10, and 2011–12 National Health and Nutrition Examination Surveys (NHANES)

	Genital						Serum						Oral				
	Women					Women				Men				Women		Men	
Demographic	03–04	05–06	07–08	09–10	11–12	03–04	05–06	07–08	09–10	03–04	05–06	07–08	09–10	09–10	11–12	09–10	11–12
All	1982	2168	2044	2209	2004	1706	2357	2101	2356	1586	2109	2084	2295	2755	2481	2747	2517
Race																	
Mexican American	459	532	432	472	227	366	608	438	505	341	542	438	517	583	275	606	313
Other Hispanic	62	78	269	248	212	65	86	289	261	56	71	252	234	329	275	295	236
White	844	861	810	950	637	797	927	846	1010	727	844	876	978	1150	762	1134	810
Black	543	591	461	403	571	416	617	440	423	387	568	412	432	517	720	563	692
Age																	
14–17	424	440	261	254	237	0	479	264	279	0	464	291	339	294	286	362	302
18–24	452	479	303	367	351	493	524	319	387	444	444	330	381	392	363	400	404
25–29	175	228	183	231	193	191	277	188	248	188	179	201	204	255	190	209	201
30–34	183	206	211	222	206	216	214	227	244	180	168	198	215	227	211	209	228
35–39	155	158	235	235	204	170	174	239	252	158	185	234	223	236	219	225	214
40–44	174	196	221	256	206	188	204	222	277	184	187	200	243	266	213	229	203
45–49	157	178	230	253	202	169	188	228	266	162	189	204	228	256	202	226	188
50–54	155	162	214	224	236	161	174	220	226	166	174	258	245	227	235	247	201
55–59	107	121	186	167	169	118	123	194	177	104	119	168	207	173	181	208	174

### Statistical analysis

Statistical analyses were performed in SAS (version 9.2). Estimates were made using two year MEC exam weights [35]. We analyzed three HPV outcomes by demographic group: infection at cervicogenital sites, infection at oral sites, and presence of HPV 6, 11, 16, or 18 antibodies in serum, which is a measure of cumulative exposure. For each pair of outcomes, we also analyzed the weighted proportion of individuals who were positive for the first outcome and positive for the second with the same HPV type (type-concordance). Analysis of cervicogenital–oral concurrence—when one person has both oral and cervicogenital infections, not necessarily of the same genotype—is left to Additional file 1.

Survey participants self-identified as Mexican American, Other Hispanic, Non-Hispanic White, Non-Hispanic Black, or Other Race - Including Multiracial (and, in 2011–12, Non-Hispanic Asian). Because of small sample sizes, we considered only the first four groups and, where indicated, combined Mexican American and Other Hispanic into one Hispanic category. In an effort to estimate vaccine efficacy and avoid confounding, seroprevalence for women in 2007–08 and 2009–10 was analyzed by vaccine status. Women reporting having had at least one dose of an HPV vaccine were considered to be vaccinated.

### Age–period–cohort modeling

Age–period–cohort (APC) models are epidemiologic models used to disentangle effects of age, period (factors affecting all people at a given time), and birth cohort (factors affecting all people born in a given time period) on prevalence (e.g. HPV prevalence) or incidence (e.g. incidence of oral cancer) [18–21, 23]. The traditional model posits that incidence rates *λ* are described by a multiplicative model with age (*A*), period (*P*), and birth cohort (*C*). This is usually treated in the logarithmic form, in which the following generalized linear model is fit:

(1) $$ \log \lambda = \beta_{0}+ \beta_{A}(A) + \beta_{P}(P) +\beta_{C}(C).  $$

A model for prevalence *P* is

(2) $$ \text{logit}~ P = \beta_{0}+ \beta_{A}(A) + \beta_{P}(P) +\beta_{C}(C).  $$

We use this model formulation for genital HPV prevalence in women and prevalence of antibodies to types 6, 11, 16, or 18 in men and women, all by race. One drawback of APC models is their inherent unidentifiability: *P*=*A*+*C*. In practice, the identifiability problem can be resolved by considering only two-effects models, typically age–period or age–cohort. In this study, age and cohort effects are modeled using splines, using five degrees of freedom/knots for both age and cohort effects, corresponding to one knot for every nine and eight years respectively. The one excpetion is cohort effects for female genital prevalence, where six knots were used, corresponding to one every nine years. APC models were fit in the statistical software R.

## Results

### Oral–cervicogenital concordance

**Fig. 1:**
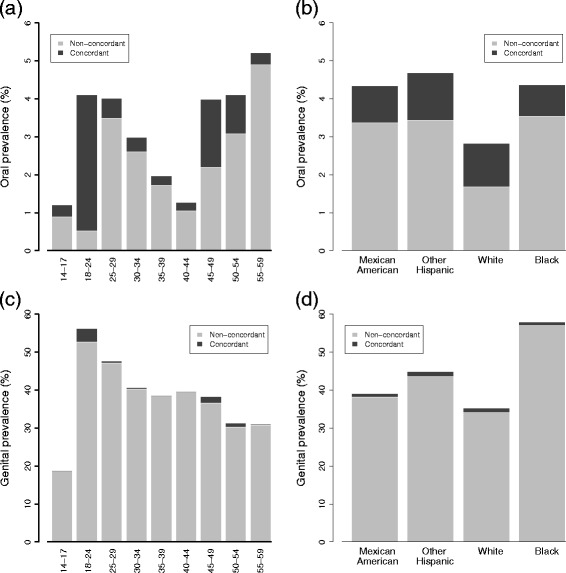
Oral and cervicogenital HPV prevalence and type-concordance for women ages 14–59. Oral (**a** and **b**) and genital (**c** and **d**) prevalence are given by age and race in 2009–10 and 2011–12 and are separated into type-concordant and non-type-concordant infections. Type-concordant infections represent a simultaneous oral and cervicogenital infection of the same genotype

Figure 1 presents stacked bar graphs of oral and cervicogenital HPV prevalence for women who were tested conclusively for both oral and cervicogenital HPV. Because oral HPV infection is relatively rare among women, we combine 2009–2010 and 2011–2012 data to support the analysis by demographic group. Prevalence at each site is broken into two categories: infections that are not type-concordant and those that are.

The percentage of oral infections among women ages 14–59 that are type-concordant with a cervicogenital infection is 33 % (1.1 % (95 % CI: 0.6–1.5) concordant infection over 3.3 % (95 % CI: 2.5–4.1) oral infection), but this varies dramatically with age. Concordance peaks at 18–24 (87 % concordant: 3.6 % (95 % CI: 1.6–5.6) over 4.1 % (95 % CI: 2.1–6.1)) and 45–49 (45 % concordant: 1.8 % (95 % CI: 0.0–3.8 %) over 4.0 % (95 % CI: 1.5–6.5)). In contrast, the vast majority of cervicogenital infections are unaccompanied by an oral infection of the same type: only 3 % of cervicogenital infections are accompanied by a concordant oral infection (1.1 % (95 % CI: 0.6–1.5) concordant infection over 39.1 % (95 % CI: 36.4–41.8) cervicogenital infection).

**Fig. 2:**
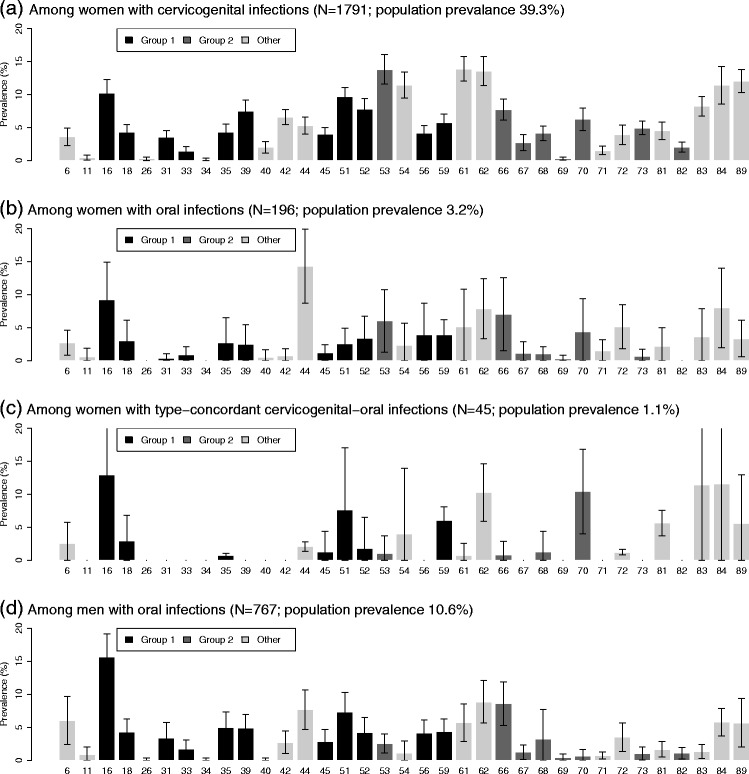
Genotype prevalence among those with HPV infections. For each category, we give the number positive for HPV, the population prevalence, and the genotype prevalence among those who have HPV. Group 1 genotypes are in dark grey, Group 2 genotypes in medium grey, and all others in light grey

In Fig. 2, we present the prevalence of genotypes in 2009–2012 among women who had (a) a cervicogenital infection, (b) an oral infection, (c) a type-concordant infection, and (d) among men who had an oral infection. Genotypes 16, 62, and 84 are common across all sites. The high prevalence of HPV 44 among oral infections may be indicative of a tropic preference for oral tissue. The high prevalence of HPV 70 and HPV 83 among oral–cervicogenital concordant infections appear to be driven by birth-cohort differences: although neither is particularly common among female oral infections when looking the overall population, HPV 83 is the second most common HPV type among women ages 18–24 (after HPV 84), the group where most oral–cervicogenital concordant infections are found, whereas HPV 70 is the third most common type among women ages 45–49 (after HPV 44 and 62), the other age group with a large fraction of concordant infections.

### Serum antibodies and concordant infections

**Fig. 3:**
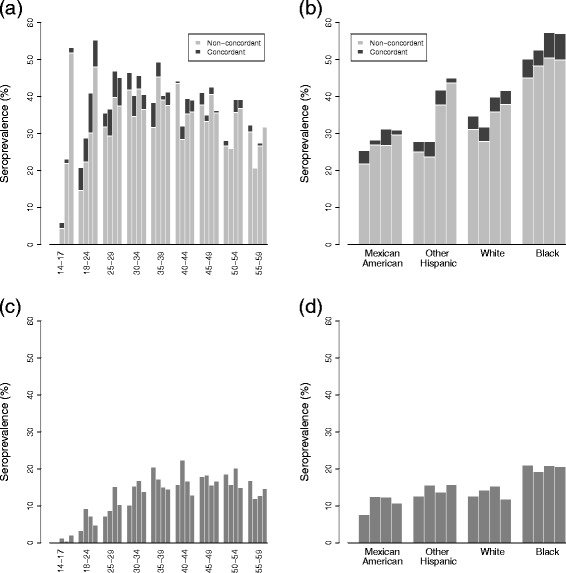
HPV seroprevalence for women, with cervicogenital type-concordance, and men, ages 14–59. Seroprevalence is for genotypes 6, 11, 16, and 18 between 2003–10 and is given (**a**) by age and (**b**) race for women and (**c**) by age and (**d**) by race for men. Type-concordance means that the antibody serotype is 6, 11, 16, or, 18 and matches the genotype of a current cervicogenital infection. The four bars in each group are 2003–04, 2005–06, 2007–08, and 2009–10 respectively. Note: Data for 14–17 year old men and women not available in 2003–04, and race data excludes ages 14–17

HPV serostatus (for types 6, 11, 16, and 18) by age and race is presented as a stacked bar graph in Fig. 3, separated by type-concordance with cervicogenital infections for women. Here, type-concordance means that an antibody serotype matches a cervicogenital genotype; this definition is a proxy for true serotype concordance because full serotype profiles are not available. Prevalence of genital infections is not available for men in this survey, and thus type-concordance in men cannot be assessed. Prevalence of type-concordance of types 6, 11, 16, and 18 among all women has remained around 10 % (2003–04: 10.4 % (95 % CI: 7.8–14.3), 2005–06: 11.1 % (95 % CI: 8.1–15.0), 2007–08: 10.3 % (95 % CI: 7.9–13.6), 2009–10: 8.4 % (95 % CI: 7.1–9.9)).

**Fig. 4:**
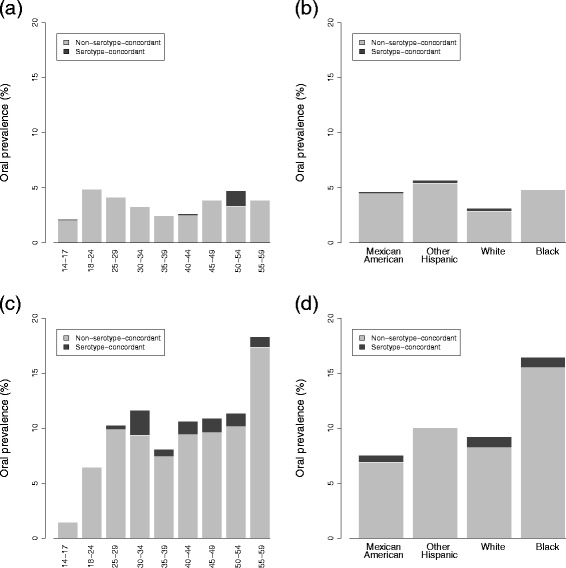
Oral HPV prevalence with serotype-concordance for men and women. Prevalence is for 2009–10 and is given (**a**) by age and (**b**) race for women and (**c**) by age and (**d**) by race for men. Serotype-concordance defined as an oral infection of type 6, 11, 16, or 18 with serum antibodies of the same type

Oral HPV prevalence by age and race is presented as stacked bar graphs in Fig. 4, with serotype-concordance, for both men and women. Serotype-concordance is defined as an oral infection of type 6, 11, 16, or 18 with serum antibodies of the same type. Prevalence of serotype-concordant oral infections is very low overall (men and women ages 14–59: 0.5 % (95 % CI: 0.2–0.8), and almost nonexistent for women (0.2 % (95 % CI: 0.0–0.8) vs 0.8 % (95 % CI: 0.4–1.3) for men). For men, this concordance remains around 10 % of the total oral infection, which varies with age; over all ages 14–59, concordance is 8.6 % of the oral prevalence (9.8 % (95 % CI: 7.9–11.7)).

### Seroprevalence and vaccination

The sharp increase in seroprevalence over subsequent surveys for 14–17 and 18–24 year old women in Fig. 3 is of particular interest. We suggest that this is the impact of vaccination. Vaccination, as expected, is associated with seroconversion: in 2009–10, 97 % (95 % CI: 93–100) of women ages 14–17 who reported at least one dose of vaccine were seropositive for type 6, 11, 16, or 18, as were 91 % (95 % CI: 85–97) of those aged 18–24.

**Fig. 5:**
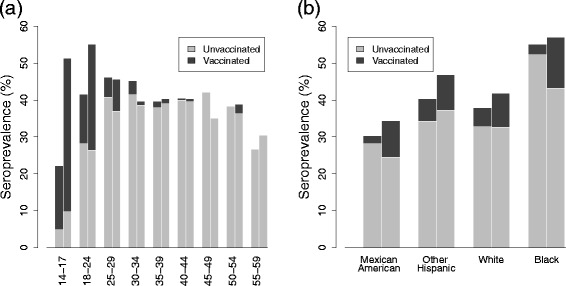
HPV seroprevalence and vaccination status for women ages 14–59. Seroprevalence is given **a**) by age and **b**) by race. The two bars in each group represent 2007–08 and 2009–10 respectively. The shaded portion of the bar gives the fraction of those who are seropositive and also reported being vaccinated. Women who reported receiving at least one dose are considered vaccinated

We present seroprevalence for women in 2007–08 and 2009–10 as stacked bar graphs with vaccination status in Fig. 5. The shaded portion of the bar gives the fraction of those who are seropositive and also reported being vaccinated. The fraction of women who are seropositive and unvaccinated (unshaded portion) in each age category is roughly the same between 2007–08 and 2009–10, and the large increase in seroprevalence among 14–17 and 18–24 year old women is seen to be the addition of vaccinated people.

Cerivcogenital HPV prevalence was also analyzed by vaccine status in 2009–12 (Table 2). Vaccination was associated with significant reduction in risk for genital infection by genotypes 6, 11, 16, and 18 in the 14–17 and 18–24 age groups. Vaccination was not associated with either an increase or decrease in risk for infection when considering all genotypes. The data were insufficient to be conclusive for the impact vaccination has on the risk of oral infection.

**Table 2:** Cerivcogenital HPV prevalence by genotype group and relative risk by vaccination status

		Unvaccinated			Vaccinated			Relative risk	
Age	Genotypes	*N*	*%* +	S.E.	*N*	*%* +	S.E.	RR	95 % CI
	All	244	17.5	2.7	233	20.1	3.5	1.1	0.7–1.8
14–17	Group 1	244	12.4	2.7	233	11.2	2.9	0.9	0.5–1.8
	6, 11, 16, 18	244	4.5	1.7	233	1.0	0.5	0.2	**0.1–0.8**
	All	448	57.7	3.4	241	56.9	3.5	1.0	0.8–1.2
18–24	Group 1	448	38.8	3.5	241	30.9	3.5	0.8	0.6–1.1
	6, 11, 16, 18	448	16.6	2.5	241	2.5	0.7	0.2	**0.1–0.3**

Prevalence is by all, oncogenic (Group 1 genotypes), and vaccine genotypes for women ages 14–17 and 18–24 in 2009–12. Here, *%*+ denotes the weighted cervicogenital HPV prevalence of the listed genotypes among the *N* people in the population. Bold relative risks intervals do not contain 1

### Age-period-cohort models

**Fig. 6:**
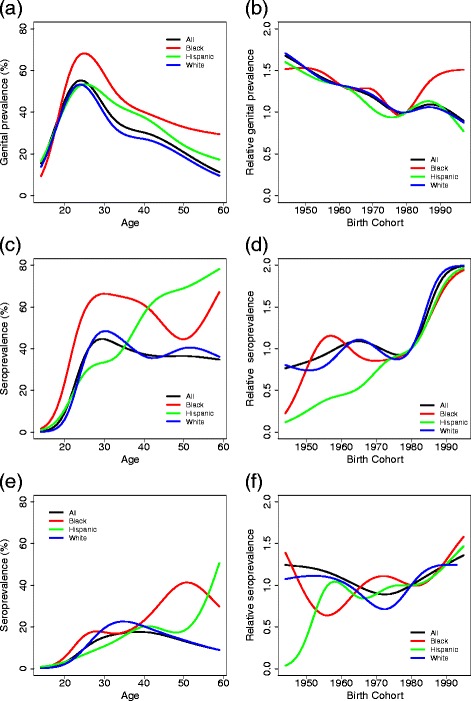
APC models of cervicogenital HPV prevalence among women, seroprevalence among women, and seroprevalence among men. Age-specific prevalence and relative prevalence by birth cohort (relative to 50 % prevalence) by race for (**a**, **b**) cervicogenital prevalence, (**c**, **d**) female seroprevalence, and (**e**, **f**) male seroprevalence

Age–cohort models fit the data better than age–period models. Figure 6 shows age-specific prevalence (relative to the 1980 birth cohort) and cohort-specific relative prevalence (relative to 50 % prevalence; for other relative prevalence, see Additional file 1) of APC models for cervicogenital prevalence (women) and seroprevalence (men and women) by race. Model fits of the data are available in Additional file 1. Here the Mexican American and Other Hispanic race categories were collapsed to Hispanic. The age-specific prevalence for Hispanic and white females is similar to that of the overall trend: peaking around age 25 and subsequently decreasing. For black women, however, prevalence is 10–20 % greater than the average. Prevalence for this demographic group also peaks slightly later. For cohort effects, the relative prevalence for all demographic groups has been decreasing since the 1940s birth cohorts, although that of black women has been increasing again since the 1980 birth cohort.

Seroprevalence for women increases dramatically between ages 20–30, after which it largely stays constant, except for Hispanic women for whom it continues to increase with age. For men, the trend is a more steady increase over the lifetime. There are strong effects on seroprevalence for birth cohorts after 1980 for women. Excluding vaccinated women in the cervicogenital analysis does not substantially affect either the age or cohort effects; excluding vaccinated women from the serum analysis does not substantially affect the age effects but does, as expected, significantly reduce the cohort effects after 1980 (results not shown).

## Discussion

The relatively low oral–cervicogenital type–concordance overall, noted by Steinau (2014) [14] and others, suggests that, although not independent, the sites have differences in natural history (e.g. time to clearance). Here we show that oral prevalence and oral–cervicogenital type-concordance vary dramatically with age. This concordance peaks for women ages 18–24, for whom nearly all oral infections are type-concordant (Fig. 1). This may suggest that many 18–24 year olds are experiencing their first oral infection and that it is either caused by autoinoculation from their genital infection or that both infections are from the same sexual partner. That we do not see the same pattern among ages 14–17 may be indicative of different sexual norms and practices between the two groups, a possibility that warrants further study. There is another age span, 45–54, with both high overall prevalence and high concordance. This may be indicative of a second sexual debut or may reflect cohort differences.

Although discussion of HPV in the public health context is often limited to mucosal types, other strains, including those that cause common and plantar warts, infect cutaneous tissue. It is possible that this tropism, that is preference for certain kinds of tissue, may be relevant within the mucosal types, with certain strains preferring genital tissue and others preferring oral tissue. It is difficult to determine whether some strains—such as types 16, 62, 84—are commonly found at both sites because of a lack of tropism, or simply because they are relatively prevalent. However, the pattern of prevalence for type 44 is consistent with a tropic preference for oral tissue. More work will be needed to assess whether this and other patterns—such as the higher prevalence of types 53, 54, and 61 among those with cervicogenital infections than among those with oral infections—can be attributed to tropism or are a reflection of cohort/timing patterns. Tropism may be important when considering an evolutionary perspective; changing sexual behaviors [36–39] may have created evolutionary pressure, assuming some level of genotype competition, favoring less tropic species.

Among those women who are seropositive for 6, 11, 16 or 18, prevalence of type-concordant cervicogenital infections is low and decreases with age, as might be expected since older individuals are more likely to have already seroconverted during previous infections. For racial demographics, seroprevalence more closely follows the cerivcogenital rather than oral profile. This finding may simply be a result of cervicogenital infections being much more common than oral ones for women, but it also raises the possibility that seroconversion may occur more readily following cervical rather than oral infection. Combined with the different prevalence of serotype-concordance for men and women (Fig. 4; the small racial deviations in the overall patterns can be attributed to low numbers and incomplete serotyping), this observation lends evidence to the theory that seroconversion is strongly site-specific and occurs primarily at mucosal epithelia. Cervicogenital infections appear to often lead to seroconversion, which, based on the lack of sero–oral type-concordance in women, may provide a defense against oral infection. Men are less likely to seroconvert from genital infections [17, 40–42], and so may be more vulnerable to oral infections, resulting in a higher oral prevalence.

Seroprevalence increases dramatically for 14–17 and 18–24 year old women over the period studied. Since the aim of the vaccine is seroconversion, it is likely that this increase is largely caused by the introduction of the vaccine (Fig. 5). Further, we want to determine the relative risk of infection given vaccination. If one does not control for age, overall cervicogenital prevalence and prevalence of oncogenic types is higher in the vaccinated population than in the unvaccinated, and prevalence of the genotypes targeted by the vaccine is only slightly lower among the vaccinated group. Because the vaccine is relatively new, it may be being given to previously infected individuals. If we restrict our attention to either 14–17 or 18–24 year olds, as in Table 2, the relative risk for any and oncogenic HPV drops to near 1, and that of the vaccine types drops to about 0.2. Our results follow those reported by Markowitz (2013) [43], who found that, in NHANES 2007–10, prevalence of vaccine types was 12.6 % among unvaccinated 14–19 year olds but 3.1 % among those vaccinated. These results suggests that the vaccine has been highly effective at preventing infections by the targeted types. The current data neither support nor refute the possibility that other genotypes may move in to fill the niche left by the vaccine genotypes.

Non-Hispanic black men and women have consistently high prevalence at genital and oral sites and seroprevalence, which is consistent with previous work [14, 32]. The APC cohort trends for cervicogenital prevalence (Fig. 6) suggest that this high prevalence relative to the other racial groups has not changed much between the 1955 and 1980 birth cohorts and has, in fact, been increasing since the 1980 birth cohort. Sexual assortativity patterns and other contributing factors may have been relatively consistent between the 1970s and 1990s, the times that people from these birth cohorts would have been mostly sexually active. The relative prevalence among women of all demographics decreased after the 1940s birth cohorts, the cohorts that experienced the sexual revolution of the 1960s. For women of all races, cohort effects for seroprevalence for women are dominated by a large increase for those born after 1980, most likely a consequence of vaccine-induced seroconversion. That age–cohort models fit better than age–period is not surprising, both as trends in STI prevalence tend to be driven by cohort sexual norms and as the NHANES time span is limited.

One strength of this study is the large sample size in the NHANES survey, which allowed analysis at a relatively fine demographic stratification. NHANES is the only population data source for oral infections and also allows for analysis of multiple sites in one individual. Additionally, the use of APC models allows for the analysis of temporal trends in the data separate from the age effects. Limitations of the NHANES data set include the relatively limited time span, especially for oral infections, the limited number of serotypes, and the lack of genital prevalence for men and anal prevalence for men and women. Although, to our knowledge, no studies have investigated seroconversion in women following anal infections, anal infections may play an important role in seroconversion in men [16, 17, 42].

## Conclusion

This study expands on the analysis by Steinau et al. [14] to provide a deeper look at oral–cervicogenital type-concordant infections. This paper is the first to analyze seroprevalence in NHANES after 2003–04 [32] and the first to look at type-concordance between serum antibodies and oral and genital infections. Our results demonstrate that with increasing vaccination coverage, seroprevalence alone is neither a good biomarker for oral infections nor a sufficient one for genital infections in women. Although not independent, infection status and serum antibodies appear insufficiently correlated to be predictive of each other. Conducting studies where sampling is done at all three sites for both men and women is paramount to fully characterize the natural history of HPV and its transmission dynamics.

This study also provides further evidence that the probability of serconversion from an infection may differ depending on which mucosal epithelium is infected. Seroconversion from cervicogenital infection appears to be common and may provide some measure of defense against oral infections, based on the lack sero-oral concordance in women. Previous studies show that men are less likely to seroconvert from genital infections than women, suggesting that they may thus be more vulnerable than women to oral infection. Further, there is some indication that some genotypes are strongly tropic, preferring to infect one site over another.

## Availability of data

NHANES data is publically available, although data on minors is restricted by the NCHS Research Data Center.

## Additional file

Additional file 1
**Supplemental material.** The supplemental material contains analysis of concurrent oral–genital infections without regard to genotype as well as further details on the statistical analyses of age–period–cohort modeling of prevalence and confidence intervals of relative risk. (PDF 164 kb)
