# Supplementary material for: Trends in HPV cervical and seroprevalence and associations between oral and genital infection and serum antibodies in NHANES 2003–2012
Source: BMC Infect Dis. 2015 Dec 21;15:575. doi: 10.1186/s12879-015-1314-0 (PMC4687319; doi:10.1186/s12879-015-1314-0)
Supplement: Additional file 1 — Supplemental material. The supplemental material contains analysis of concurrent oral–genital infections without regard to genotype as well as further details on the statistical analyses of age–period–cohort modeling of prevalence and confidence intervals of relative risk. (PDF 164 kb) [file 12879_2015_1314_MOESM1_ESM.pdf]

Supplementary material

Oral–cervicogenital concurrence

Overall oral–cervicogenital concurrence and type-concordance was previously examined by Steinau (2014), who reported that the relative risk for women ages 18–59 for having an oral infection given a cervicogenital infection was 4.86 in 2009–10. We report that relative risk is 5.3 for women ages 14–59 in 2009–12, and that the relative risk for having an oral infection given a genital infection changes dramatically with age, as seen in Table S1. Indeed, a large percentage of oral infections are accompanied by a cerivcogenital infection. Over 2009–2012, 77% of oral infections among women ages 14–59 were concurrent with a cervicogenital infection (2.5% (95%CI: 1.9–3.2) concordant infection over 3.3% (95%CI: 2.5–4.1) oral infection). In Figure S1, we see that percent concurrence varies with age.

In contrast, the vast majority of cervicogenital infections are unaccompanied by an oral infection of the same type: only 7% of cervicogenital infections are accompanied by an oral infection (2.5% (95%CI: 1.9–3.2) concordant infection over 39.1% (95%CI: 36.4–41.8) genital infection). The relative risk of having a cervicogenital infection given an oral infection is only 2.0 and only varies slightly by demographic, as seen in Table S2.

Age–Period–Cohort modeling

Given an incidence I and at-risk population N, it is straightforward to model $\lambda = I/N$. If, alternatively, one wishes to model prevalence P, one may convert as follows.

$$\log \lambda = \log \frac{I}{N} = \log \frac{\frac{I}{I+N}}{\frac{N}{I+N}} = \log \frac{P}{1-P} = \text{logit } P$$

If, as in the main text, we model age and cohort only, we have

$$\text{logit } P = \beta_A(A) + \beta_C(C).$$

We identify age effects with $\theta_A = \exp(\beta_A(A))$ and cohort effects with $\theta_C = \exp(\beta_C(C))$. Then, age–cohort specific prevalence is given by

$$P(A, C) = \frac{\theta_A \theta_C}{1 + \theta_A \theta_C}.$$

Age-specific prevalence at the reference cohort, as found in the main text (Figure 5a, c, e) is found when $\theta_C = 1$. The relative prevalence by cohort relative to P is given by $\frac{\theta_C}{1+P(\theta_C - 1)}$; plots for $P = 0.5$ are presented in the main text (Figure 5b, d, f). For completeness, we plot the raw age and cohort effects (Figure S2) and the model fits to the data (Figures S3, S4, S5).

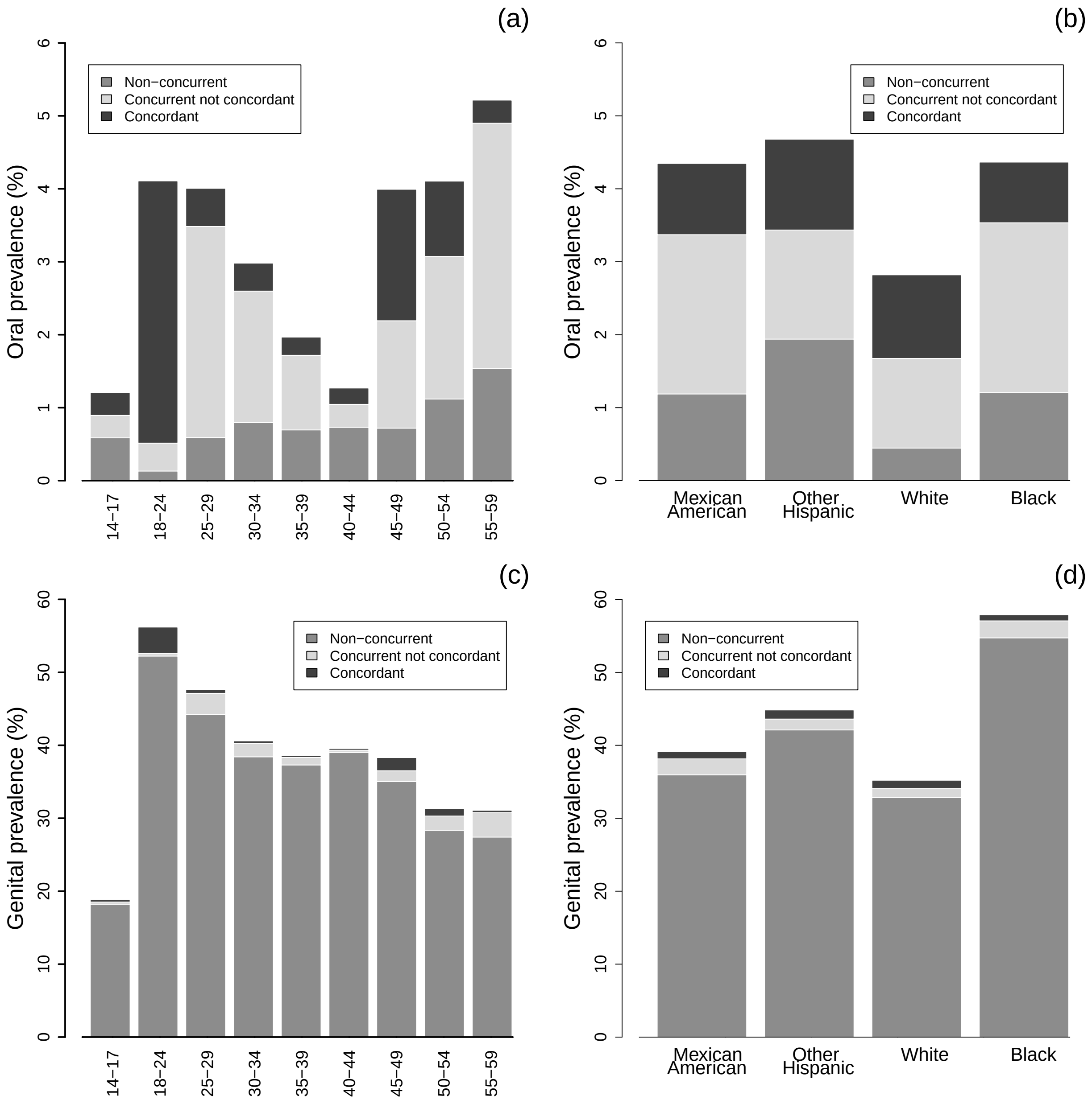

Figure S1: **Oral and cervicogenital HPV prevalence, concurrence and type-concordance for women ages 14–59**. Oral (a and b) and cervicogenital (c and d) prevalence are given by age and race in 2009–10 and 2011–12 and are separated into type-concordant, concurrent but not concordant, and non-current infections.

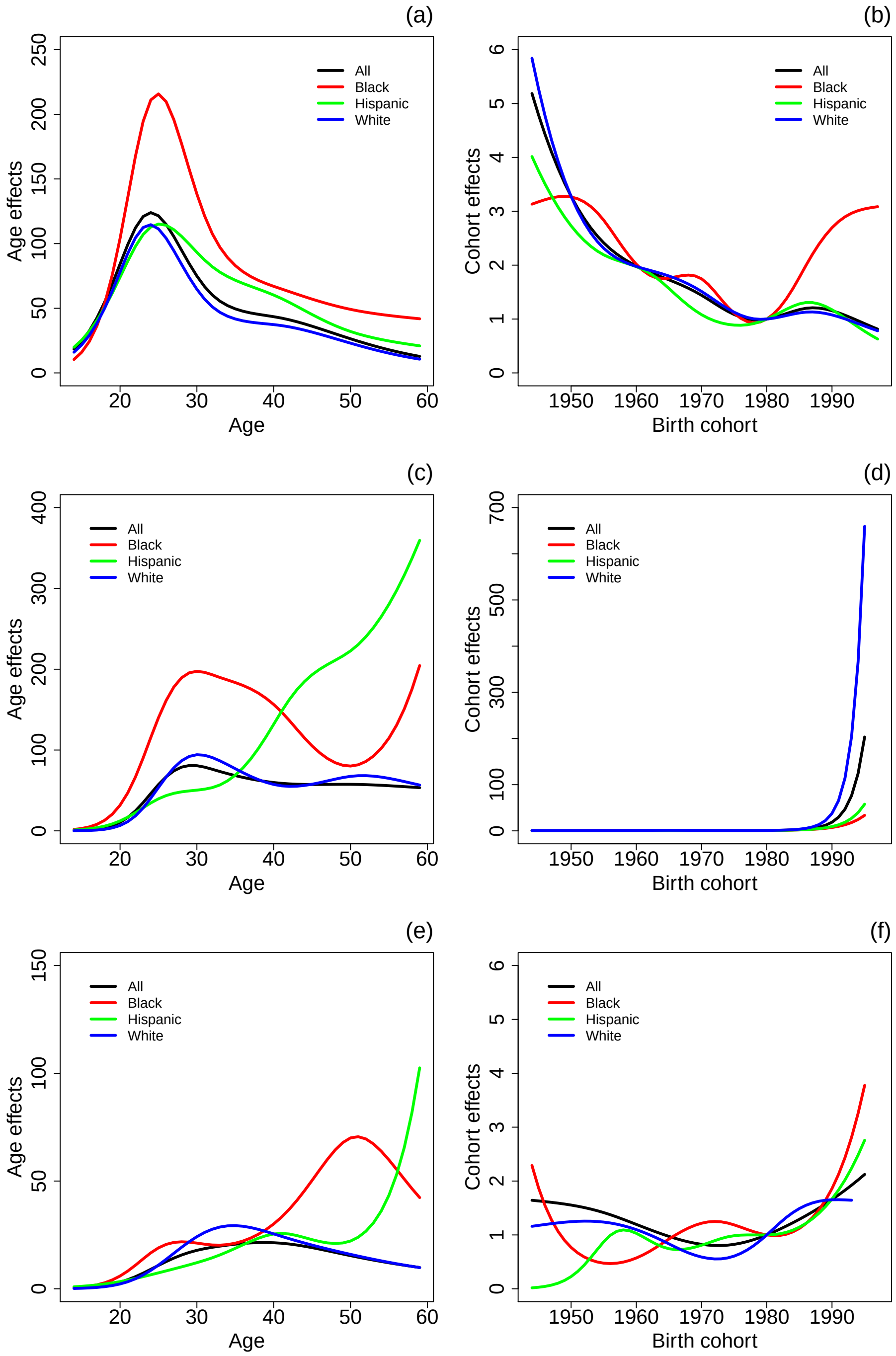

Figure S2: **Age and cohort effects for cervicogenital prevalence and seroprevalence among women and men.** Age effects and cohort effects by race for (a,b) cervicogenital prevalence, (c,d) female seroprevalence, and (e,f) male seroprevalence.

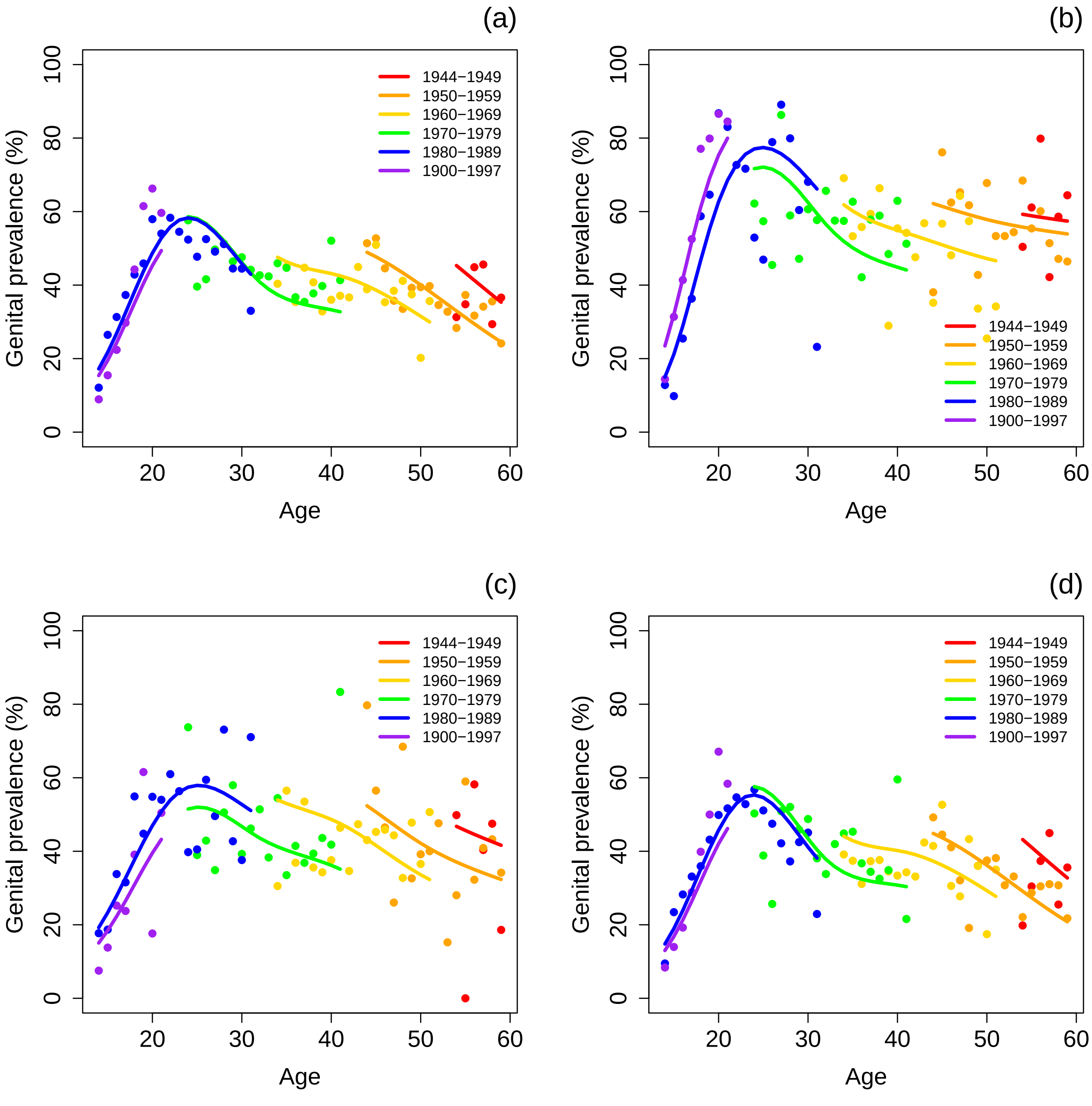

Figure S3: **Age-specific cervicogenital prevalence by birth cohort with APC model.** Prevalence is given for (a) all women, (b) black women, (c) Hispanic women, and (d) white women.

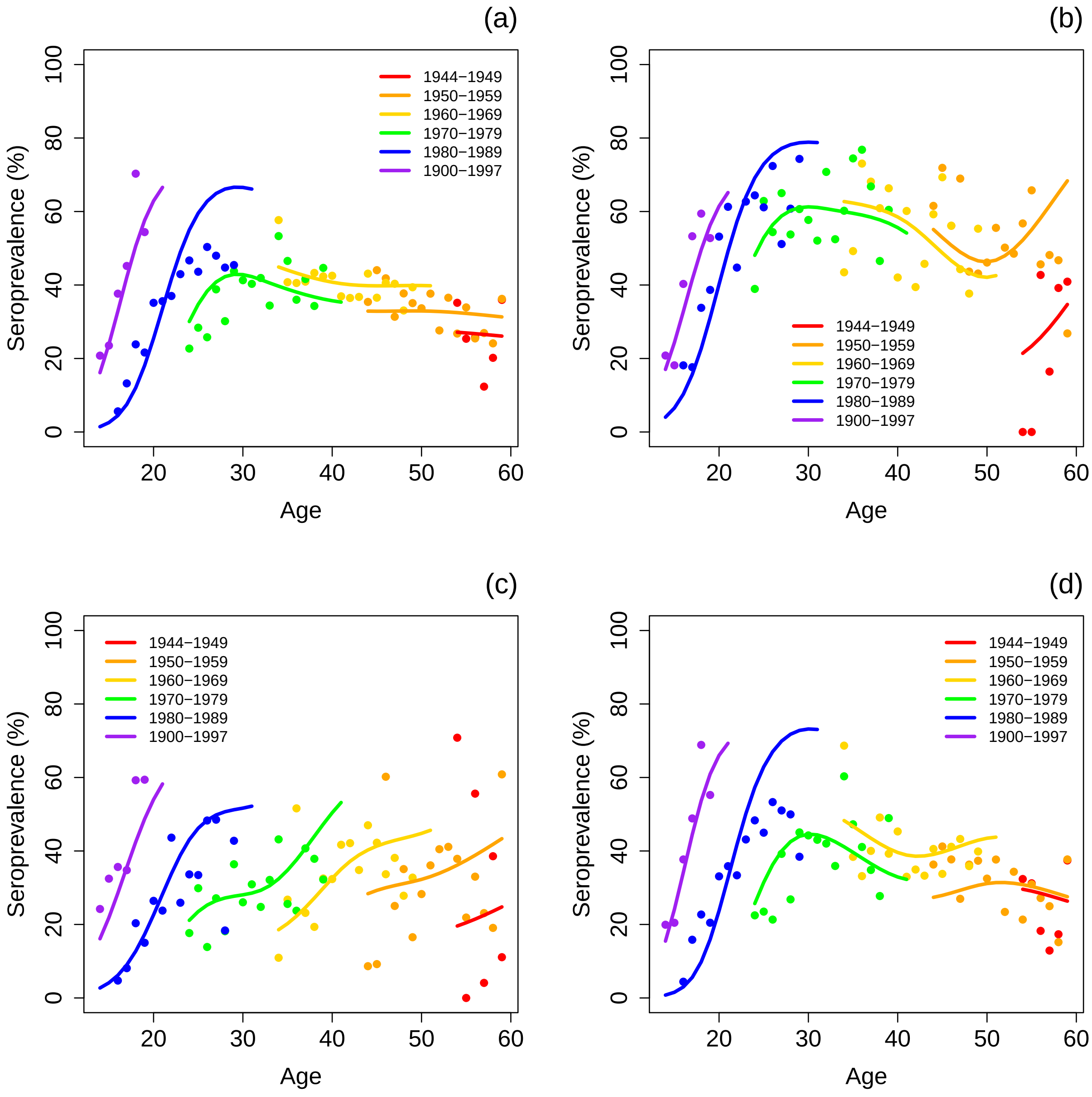

Figure S4: **Age-specific seroprevalence for women by birth cohort with APC model.** Prevalence is given for (a) all women, (b) black women, (c) Hispanic women, and (d) white women.

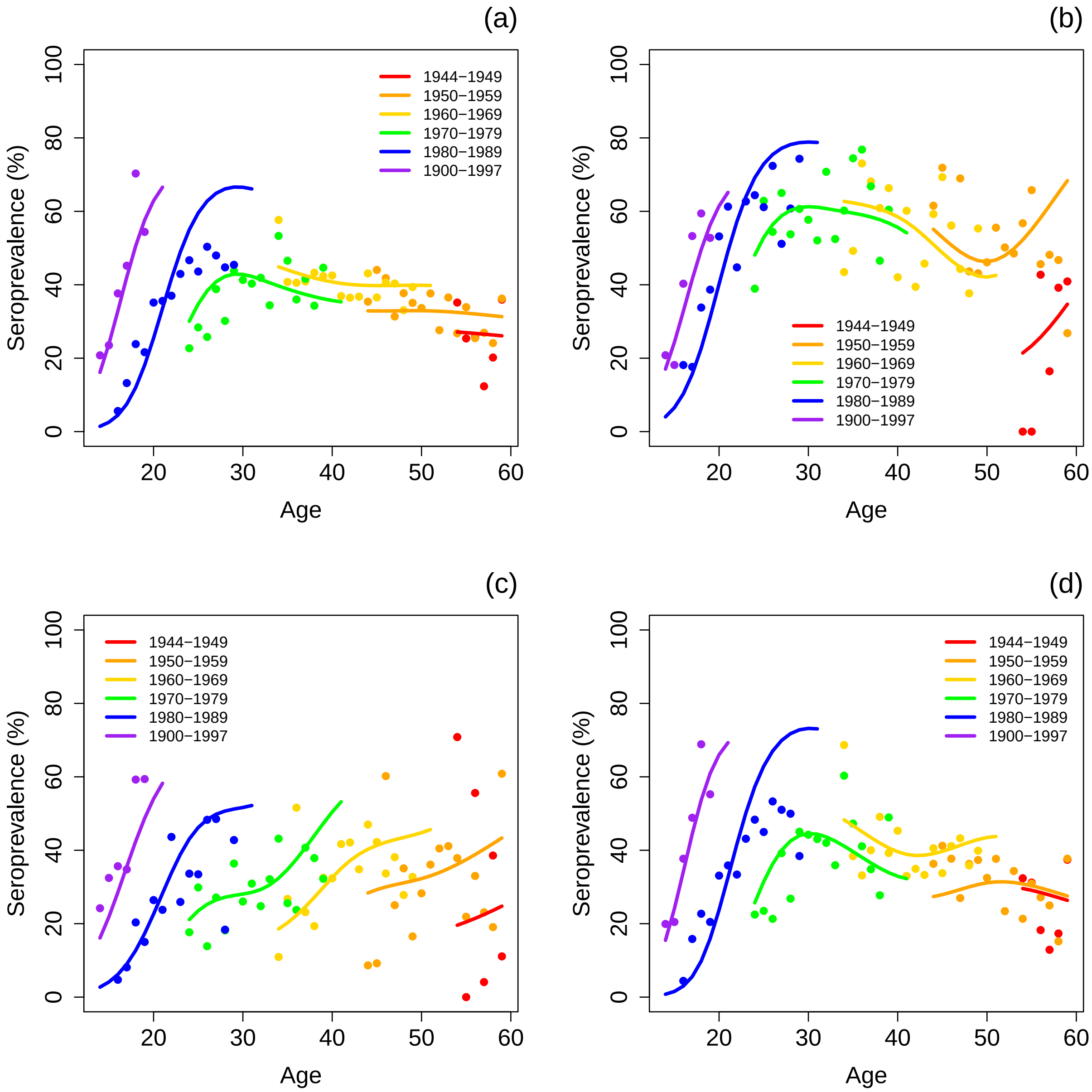

Figure S5: **Age-specific seroprevalence for men by birth cohort with APC model.** Prevalence is given for (a) all men, (b) black men, (c) Hispanic men, and (d) white men.

Confidence intervals for prevalence ratios

Confidence intervals for prevalence ratios were calculated by a log transformation. The standard errors of the natural log of prevalence ratios were approximated as follows:

$$SE(\ln \hat{p}_1/\hat{p}_0) = \sqrt{\frac{\hat{\sigma}^2_{\hat{p}_1}}{\hat{p}^2_1} + \frac{\hat{\sigma}^2_{\hat{p}_0}}{\hat{p}^2_0}}$$

Table S1: **Oral HPV prevalence and relative risk for women ages 14–59 by +/- cervicogenital HPV status.** Prevalence is for 2009–12. Here, %+ gives the weighted HPV oral prevalence among the N people in the given population. Bold relative risks intervals do not contain 1.

	Oral Prevalence Among Genital +			Oral Prevalence Among Genital –			Relative Risk	
Demographic	N	%+	S.E.	N	%+	S.E.	RR	95%CI
All	1665	6.5	0.8	2275	1.2	0.2	5.3	**3.4–8.4**
Race								
Mexican American	244	8.1	2.0	276	2.0	0.8	4.1	**1.6–10.7**
Other Hispanic	182	6.1	1.6	247	3.5	0.9	1.7	0.8–3.6
White	586	6.7	1.4	897	0.7	0.3	9.8	**4.0–23.5**
Black	522	5.5	1.1	388	2.9	0.8	1.9	1.0–3.8
Age								
14–17	97	3.2	1.1	380	0.7	0.4	4.5	**1.4–15.1**
18–24	385	7.1	1.7	298	0.3	0.2	23.6	**5.5–101**
25–29	206	7.2	2.5	191	1.1	0.5	6.3	**2.0–20.4**
30–34	166	5.4	1.8	222	1.3	0.7	4.0	**1.2–13.4**
35–39	174	3.3	1.5	237	1.1	0.3	2.9	1.0–8.6
40–44	177	1.4	0.7	251	1.2	0.9	1.1	0.2–6.4
45–49	179	8.5	2.9	245	1.2	0.5	7.3	**2.4–22.4**
50–54	159	9.5	3.5	262	1.6	0.8	5.8	**1.8–19.4**
55–59	122	11.8	4.4	189	2.2	1.0	5.3	**1.7–16.6**

Table S2: **Cervicogenital HPV prevalence and relative risk for women ages 14–59 by +/- oral HPV status.** Prevalence is for 2009–12. Here, %+ gives the weighted HPV cerivcogenital prevalence among the the N people in the given population. Bold relative risks do not contain 1.

	Genital Prevalence Among + Oral			Genital Prevalence Among - Oral			Relative Risk	
Demographic	N	%+	S.E.	N	%+	S.E.	RR	95%CI
All	150	77.4	3.6	3790	37.8	1.3	2.0	**1.8–2.3**
Race								
Mexican American	29	72.7	8.8	621	37.6	2.0	1.9	**1.5–2.5**
Other Hispanic	19	58.6	9.7	410	44.2	2.4	1.3	0.9–1.9
White	52	84.2	5.6	1431	33.8	1.6	2.5	**2.1–2.9**
Black	38	72.4	5.2	872	57.3	2.0	1.3	**1.1–1.5**
Age								
14–17	9	51.3	14.3	468	18.4	1.8	2.8	**1.6–5.0**
18–24	29	96.8	2.4	654	54.5	2.8	1.8	**1.6–2.0**
25–29	17	85.2	7.6	380	46.1	3.0	1.8	**1.5–2.3**
30–34	16	73.3	14.3	372	39.6	3.0	1.9	**1.2–2.8**
35–39	11	64.7	12.9	400	38.1	2.7	1.7	**1.1–2.6**
40–44	8	42.4	22.0	420	39.5	3.3	1.1	0.4–3.0
45–49	20	82.0	8.0	404	36.5	3.1	2.2	**1.7–2.9**
50–54	22	72.7	11.4	399	29.6	4.6	2.5	**1.6–3.8**
55–59	18	70.5	12.8	293	28.9	3.5	2.4	**1.6–3.7**
